# Hydrotherapy for the Treatment of Pain in People with Multiple Sclerosis: A Randomized Controlled Trial

**DOI:** 10.1155/2012/473963

**Published:** 2011-07-14

**Authors:** Adelaida María Castro-Sánchez, Guillermo A. Matarán-Peñarrocha, Inmaculada Lara-Palomo, Manuel Saavedra-Hernández, Manuel Arroyo-Morales, Carmen Moreno-Lorenzo

**Affiliations:** ^1^Department of Nursing and Physical Therapy, University of Almeria (UAL), Carretera de Sacramento s/n, 04120 Almería, Granada, Spain; ^2^Health District Granada, Andalusian Health Service, 18012 Granada, Spain; ^3^Department of Physical Therapy, University of Granada (UGR), Spain

## Abstract

*Background*. Multiple sclerosis (MS) is a chronic demyelinating neurological disease. Several studies have reported that complementary and alternative therapies can have positive effects against pain in these patients. *Objective*. The objective was to investigate the effectiveness of an Ai-Chi aquatic exercise program against pain and other symptoms in MS patients. *Methods*. In this randomized controlled trial, 73 MS patients were randomly assigned to an experimental or control group for a 20-week treatment program. The experimental group underwent 40 sessions of Ai-Chi exercise in swimming pool and the control group 40 sessions of abdominal breathing and contraction-relaxation exercises in therapy room. Outcome variables were pain, disability, spasm, depression, fatigue, and autonomy, which were assessed before the intervention and immediately and at 4 and 10 weeks after the last treatment session. *Results*. The experimental group showed a significant (*P* < 0.028) and clinically relevant decrease in pain intensity *versus* baseline, with an immediate posttreatment reduction in median visual analogue scale scores of 50% that was maintained for up to 10 weeks. Significant improvements were also observed in spasm, fatigue, disability, and autonomy. *Conclusion*. According to these findings, an Ai-Chi aquatic exercise program improves pain, spasms, disability, fatigue, depression, and autonomy in MS patients.

## 1. Introduction

Multiple sclerosis (MS) is a chronic demyelinating neurological disease afflicting young and middle-aged adults that impairs coordination, strength, cognition, and sensation [1]. Although treatment with immunomodulatory agents can affect the course of MS, it is not currently curable [2]. It is the most frequent disabling neurologic disease among young and middle-aged adults in North America and Europe [3].

Patients with MS often request complementary and alternative medicine (CAM) in different forms, but the effectiveness of these therapies has not been demonstrated in MS patients [4]. A recent study found that 50–75% of patients with MS used CAM because it reduces the severity of painful symptoms and offers functional improvement [1, 5]. Many MS patients reported that they turned to CAM due to dissatisfaction with conventional pharmacological therapies and experienced a considerable improvement in symptoms as a result [4–6]. However, although CAM is widely used by MS patients, there is no scientific evidence to support its effectiveness [5, 6]. The majority of MS patients use CAM alongside their conventional treatment and report that they receive a benefit from these alternative therapies [4]. Survey results suggest that MS patients choosing to use both CAM and conventional medicine integrate both types of medicine to attain a more holistic healthcare approach [3]. It is well known that female MS patients and those with a higher education and income are more likely to use CAM [5, 7, 8]. The severity of the disease may also influence the use of CAM [9, 10]. MS patients appear to especially value mind-body therapies, perhaps attributable to their psychological effects in reducing stress, which is known to exacerbate MS symptoms [5, 11, 12]. 

 Recent guidelines from the National Institute of Health and Clinical Excellence (NICE) affirmed that MS patients should be informed of findings on the benefits of certain approaches but declared that insufficient evidence is available to make a firmer recommendation [13]. Named techniques include reflexology, massage, fish oils, magnetic field therapy, neural therapy, massage plus body work, Tai-Chi, and multi-modal therapy [14]. MS patients also report the therapeutic use of exercise, vitamins, herbal and mineral supplements, relaxation techniques, acupuncture, cannabis, and massage, mainly for the treatment of pain, fatigue, and stress [15]. Maloni [16] reported that Tai-Chi, meditation, and hypnotherapy may improve the quality of life and reduce pain in MS patients by interfering with pain conduction, producing analgesia through nociceptive pathways.

 Aquatic exercise can refer to pool therapy, hydrotherapy, or balneotherapy [17]. Hydrotherapy is frequently applied to patients with painful neurological or musculoskeletal alterations, [18] because the heat and floatability of the water can block nociceptors by acting on thermal receptors and mechanoreceptors and exert a positive effect on spinal segmental mechanisms [19]. Warm water can also increase the blood flow, helping to dissipate allogeneic chemicals and enhance muscle relaxation. Finally, the hydrostatic effect of water can alleviate pain by reducing peripheral edema and sympathetic nervous system activity [17, 20]. A systematic review on crenobalneotherapy in patients with limb osteoarthritis found that it reduced pain and improved function and quality of life [21]. CAM is frequently used in spa therapy *in situ* without exercise for various chronic diseases, with highly positive effects in middle-aged and elderly patients [22, 23]. The main aim of this paper was to determine the effectiveness of hydrotherapy to modify pain, quality of life, and other symptoms in MS patients.

## 2. Materials and Methods

We performed an experimental clinical trial with a control group (MS patients receiving relaxation exercise protocol in therapy room) and experimental group (MS patients undergoing an Ai-Chi exercise protocol in swimming-pool). The study period was from January 1 2009 through June 30 2010. 

### 2.1. Participants

Study subjects were MS patients from the Multiple Sclerosis Association of Almeria (AEMA) in Spain. Initial screening included medical history and pretrial questionnaire, gathering data on age, time since diagnosis, course of the disease, and Expanded Disability Status Scale (EDSS). Inclusion criteria were MS diagnosis, age between 18 and 75 yrs, Visual Analogue Scale (VAS) pain score >4 for at least two months, and EDSS ≤7.5. Exclusion criteria were treatment with another CAM, either current or within the previous 3 months, and relapse requiring hospitalization or steroid treatment within the past 2 months. 

Among the 198 accessible patients, 98 did not meet the inclusion criteria and 27 refused participation in the study. The selection of groups was balanced for the type of medication received, using a stratification system that generates a sequence of letters (from a table of correlatively ordered permutations) for each combination of categories. Each patient was assigned a sequence of letters according to the type of medication they were receiving, and the different sequences were placed in sealed envelopes that were randomly assigned to each study group.

Informed consent was obtained from each patient before entering the study, which was performed in accordance with the Helsinki Declaration (2008 modification) on research projects and with national legislation on clinical trials (Law 223/2004 6 February), biomedical research (Law 14/2007 3 July), and patient confidentiality (Law 15/1999, 13 December). The study was approved by the ethics and research committee of the University of Almeria.

### 2.2. Procedure

The patients were randomly assigned by a blinded researcher, using a computer-generated randomized list, to an Ai-Chi exercise group (*n* = 36) or relaxation exercise group (*n* = 37). Both groups received treatment sessions twice a week for 20 weeks, on Mondays and Thursdays for the experimental group and on Tuesdays and Fridays for the control group. Power calculations were carried out after 20 patients had been treated, estimating a minimal sample size of 33 participants per group for a power of 80% and standard deviation (SD) of 3.1.

### 2.3. Intervention

The Ai-Chi exercise program was conducted in a swimming pool with a water temperature of 36°C. Patients took a shower with a water temperature of 35.5°C before entering the pool. The air temperature was maintained at 20°–25°C. A single physiotherapist led all of the Ai-Chi exercise sessions, teaching the 16 movements that constitute this therapy (which requires no additional material). There was a maximum of 10 participants per session. Ai-Chi exercises, all performed in shoulder-depth water, use a combination of deep breathing and slow, broad movements of the arms, legs, and torso to work on balance, strength, relaxation, flexibility, and breathing. The 16 movements or postures are designated as follows: contemplating, floating, uplifting, folding, soothing, gathering, freeing, transferring, accepting, accepting with grace, rounding, flowing, relaxing, and sustaining. Relaxation is induced by the slow and wide movements of arms and legs and by the focus on the breathing [24]. The principles of Ai-Chi are *Yuan* (circular movements seeking internal and external harmony), *Sung* (internal and external relaxation to promote blood circulation), *Ching* (absence of tension in the body), *Yun* (movement at a given speed that is always controlled by the mind), *Cheng* (correct maintenance of balance and posture), *Shu* (easy, comfortable, and relaxed movement of the body), and *Tsing* (direction of thought towards the mind, concentration). Relaxing Tai-Chi music (album by Oliver Santi & Friends) was played to the participants during the sessions, which lasted 60 minutes, beginning and ending with 10 minutes of relaxation in the water [24]. Throughout these 10 min relaxation periods, the patients performed abdominal breathing simultaneous with contraction-relaxation exercises of muscle groups in hands, arms, shoulders, face, neck, thighs, legs, and feet while standing in the shoulder-depth water [25].

The same physiotherapist also led the exercise sessions for the control group, which were conducted in a therapy room at a temperature of around 26°C. The patients underwent the same exercise program followed by the experimental group during the relaxation periods (abdominal breathing plus guided contraction-relaxation) but in supine position on an exercise mat (tatami). No ambient music was played during these control sessions.

### 2.4. Outcome Assessment

Both groups were evaluated pre-treatment (baseline) and immediately and 4 and 10 weeks after the treatment period by a researcher blinded to group allocation. Primary measurement variables were pain, using a VAS (range, 0–10 points) and the Pain Rating Index (PRI) (0–77 points) and Present Pain Intensity (PPI) (0–5) from the McGill Pain Questionnaire (MPQ) [26], and disability, using the Roland Morris Disability Questionnaire (RMDQ) (0–24) [27]. Secondary measurement variables were the scores for: spasm VAS (0–10), Multiple Sclerosis Impact Scale-29 (0–100) [28], Modified Fatigue Impact Scale (physical score 0–36, cognitive score 0–40, psychosocial score 0–40) [29], Fatigue Severity Scale (1–7), [30] Becks Depression Inventory (0–63) [31], and Barthel Index (0–100 points) [32]. 

SPSS version 18.0 (SPSS Inc., Chicago IL) was used for the data analyses. After a descriptive study of the demographical variables, the distribution of variables was analyzed by means of the Kolmogorov-Smirnof test. An imputed score was calculated for standardized scales missing ≤10% of responses. Independent *t*-tests were used to compare baseline demographic characteristics between participants and dropouts and between experimental and control groups (randomization test).

Changes in scores for anxiety, pain, depression, quality of sleep, and quality of life were analyzed by using a 2 (Groups: experimental and placebo) × 4 (Time points: baseline and immediately and 4 and 10 weeks after the treatment period) repeated-measures analysis of variance (ANOVA). A Student's *t*-test for paired measures was used to determine the effectiveness of treatments. Differences between study groups were analyzed with a Student's *t-*test for independent samples. *P* < 0.05 was considered significant in all tests.

## 3. Results

**Figure 1:**
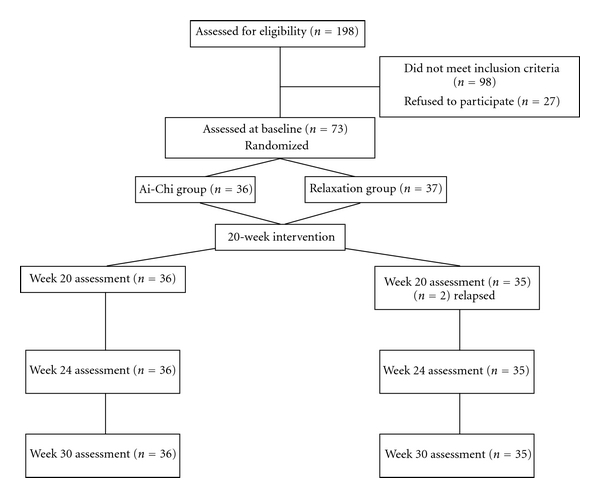
Recruitment and progress of participants through the trial.

Out of the 198 initially screened patients, 27 refused participation and 98 failed to meet the inclusion criteria due to score <4 in pain VAS, predicted drug regimen changes during the study, family problems, transport difficulties, or leaving the region. Two patients in the control group were excluded during the 20-week treatment period due to relapse. The final study sample comprised 36 patients in the experimental group (26 females) and 35 in the control group (24 females). The flow of patients through the study is depicted in Figure 1.

 At baseline, the groups did not significantly differ in demographic characteristics (Table 1) or any measurement variables (Table 2). Patients most frequently reported their worst pain to be musculoskeletal back pain (51 with lumbar pain and 22 with cervical pain), followed by pain in legs (*n* = 32), feet (*n* = 29), arms (*n* = 23), shoulders (*n* = 17), and forearms (*n* = 13). No significant differences in anatomic pain distribution were found between the groups. 

### 3.1. Primary Outcome Measures

#### 3.1.1. Pain VAS

**Figure 2:**
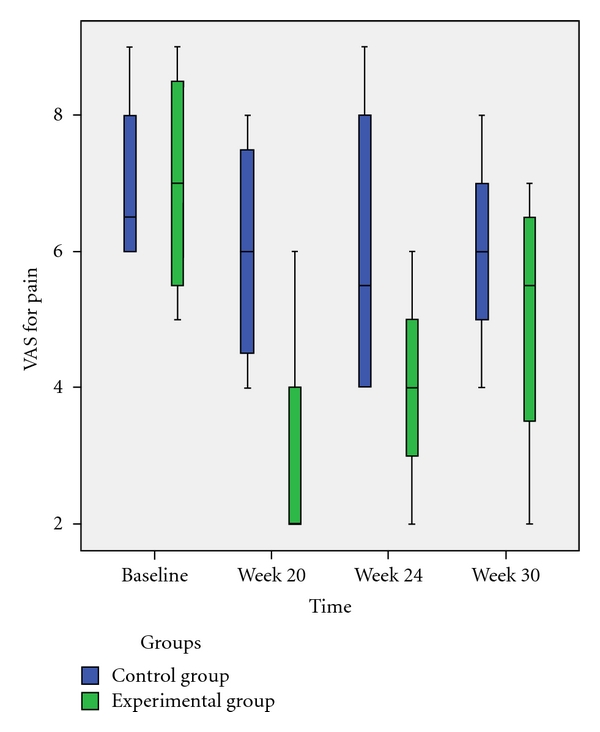
Mean Pain Visual Analogue Scale scores by groups at different time points. Values are presented as means with error bars.

Immediately after the final treatment session (week 20 after treatment onset), the experimental group showed a significant reduction in pain VAS score *versus* baseline (*P* < 0.028), with a 50% reduction in pain levels (Figure 2); the pain continued to be significantly lower *versus* baseline at weeks 24 (*P* < 0.035) and 30 (*P* < 0.047). The groups significantly differed in pain VAS score at weeks 20 (*P* < 0.044) and 24 (*P* < 0.049). The control group showed no significant differences in pain VAS score *versus* baseline at any time point.

#### 3.1.2. MPQ PRI

The experimental group showed a significant pain reduction at weeks 20 (*P* < 0.037) and 24 (*P* < 0.043) *versus* baseline, and the groups significantly differed in PRI scores (*P* < 0.029) at these time points (*P* < 0.044) and (*P* < 0.031), respectively (Table 2). At week 30, the experimental group no longer showed a significant pain reduction *versus* baseline. The control group showed no significant difference with baseline PRI scores at any time point.

#### 3.1.3. MPQ PPI

The experimental group showed a significant reduction (*P* < 0.034) in PPI at week 20. The control group showed no significant difference with baseline PPI scores at any time point, and no significant difference between the groups was observed at any time point (Table 2).

#### 3.1.4. RMDQ

Significant decreases in RMDQ scores were found in both groups at weeks 20 (*P* < 0.021, experimental group; *P* < 0.033, control group) and 24 (*P* < 0.026, experimental group; *P* < 0.048, control group) and in the experimental group alone at week 30 (*P* < 0.028). Significant differences between groups were found at weeks 20 (*P* < 0.044), 24 (*P* < 0.042), and 30 (*P* < 0.027) (Table 2).

### 3.2. Secondary Outcome Measures

#### 3.2.1. Spasm VAS

The experimental group showed a significant decrease in spasm VAS score at week 20 (*P* < 0.039) that was maintained at week 24 (*P* < 0.040) but not at week 30 (*P* < 0.067 versus week 20). The control group showed no significant difference *versus* baseline at any time point. The groups significantly differed in spasm VAS at weeks 20 (*P* < 0.048) and 24 (*P* < 0.042) (Table 2). 

#### 3.2.2. Multiple Sclerosis Impact Scale-29

**Psychological**

Both groups showed a significant reduction at week 20 (*P* < 0.009, experimental group; *P* < 0.046, control group) that was maintained in the experimental group at weeks 24 (*P* < 0.018) and 30 (*P* < 0.024). There were significant differences between study groups at weeks 20 (*P* < 0.023), 24 (*P* < 0.027), and 30 (*P* < 0.038) (Table 2).

**Physical**

The experimental group showed a significant score reduction at week 20 (*P* < 0.013) that was maintained at weeks 24 (*P* < 0.017) and 30 (*P* < 0.025). The control group showed no significant difference *versus* baseline at any time point; only 6% of patients in the control relaxation program evidenced a score improvement. There were significant differences between study groups at weeks 20 (*P* < 0.014), 24 (*P* < 0.019), and 30 (*P* < 0.027) (Table 2).

#### 3.2.3. Modified Fatigue Impact Scale

**Physical**

The experimental group showed a significant score reduction at week 20 (*P* < 0.032) that was maintained at week 24 (*P* < 0.038). The control group showed no significant differences *versus* baseline, and only 9% evidenced an improvement. The groups significantly differed at weeks 20 (*P* < 0.042) and 24 (*P* < 0.044). An improvement was shown by 48% of the experimental group (Table 2).

**Cognitive**

The experimental group showed a significant reduction at week 20 (*P* < 0.038) that was maintained at week 24 (*P* < 0.044). The control group showed no significant differences *versus* baseline and no significant difference was observed between groups at any time point (Table 2).

**Psychological**

The experimental group showed a significant score reduction at weeks 20 (*P* < 0.041) and 24 (*P* < 0.038). The control group showed no significant differences *versus* baseline at any time point, with 27% evidencing an improvement. No significant difference was observed between groups at any time point.

#### 3.2.4. Fatigue Severity Scale

The experimental group showed a significant reduction in fatigue at weeks 20 (*P* < 0.043) and week 24 (*P* < 0.046). The control group showed no significant differences *versus* baseline at any time point, with 12% evidencing an improvement. The groups significantly differed in scores at week 24 (*P* < 0.048).

#### 3.2.5. Beck Depression Inventory II

The experimental group showed a significant reduction at weeks 20 (*P* < 0.028) and 24 (*P* < 0.040). The control group showed no significant differences *versus* baseline at any time point. The groups significantly differed in scores at weeks 20 (*P* < 0.031) and 24 (*P* < 0.039).

#### 3.2.6. Barthel Index

The experimental group showed a significant reduction at weeks 20 (*P* < 0.047) and 24 (*P* < 0.049). The control group showed no significant differences *versus* baseline at any time point, with 2% evidencing an improvement. No significant difference was found between the groups at any time point.

## 4. Discussion

In this randomized controlled clinical trial, a twice-weekly 20-week Ai-Chi aquatic exercise program significantly reduced pain levels in MS patients and improved fatigue, spasms, depression, and quality of life, with no adverse effects. These beneficial effects lasted for 4 and 10 weeks after the end of the program and were superior to those obtained by control MS patients after an exercise program in a therapy room. These first results on the effectiveness of Ai-Chi to treat pain in MS patients are in agreement with findings of pain reduction and mobility improvement in other patient populations undergoing this exercise therapy [17, 33, 34]. 

 Spasticity, which has a major impact on overall disability in MS [35], is considerably improved by aquatic exercise, because patients are able to perform wider voluntary movements while immersed in water [17, 35]. The resulting increase in exercise level also has a positive impact on fatigue [36]. The provision of ambient music may have contributed to the positive effects of the Ai-Chi sessions, by increasing motivation and distracting participants from any discomfort produced by the physical exercise [36]. Music promotes natural rhythmic movements in the water, enhancing mobility, and exercise with musical stimulus can influence oscillators and timekeeper functions of the brain [37]. 

 One systematic review found exercise training to be associated with an improvement in activity-related and walking mobility in MS patients [34, 38]. Exercise is associated with physical and psychological health benefits and a reduced risk of cardiovascular disease, diabetes, depression, and cancer [39]. Individuals with MS should be encouraged to engage in exercise as an adjuvant therapy to mitigate progressive mobility impairment, especially given the prevalence of physical inactivity among these patients. The exercise activity in the Ai-Chi aquatic program offers benefits in the treatment of neural and musculoskeletal diseases that may not be obtained in hydrotherapy modalities with only passive immersion, for example, balneotherapy [17]. The experience of mobility improvement through this exercise program can be exploited by clinicians to promote an active lifestyle and develop strategies to enhance their physical activity in patients with MS. Exercise therapy improves mobility in all types of MS, especially in secondary-progressive and primary-progressive MS, in which pharmacological treatment is minimally effective to improve mobility and reduce disease progression [34, 40, 41]. 

 A recent study [24] comparing the effects of a 6-week Ai-Chi aquatic exercise program and stretching exercises in fibromyalgia patients demonstrated a clinically significant reduction in pain and quality of sleep that lasted 4 and 12 weeks after the end of the program. However, the authors found no evidence of clinical benefits in depression, fatigue, or mental health. In contrast, our longer Ai-Chi program achieved a significant reduction in depression and fatigue in the present experimental group. Aquatic exercise programs ranging from 3 weeks to 12 months have been studied, but elevated dropout rates have been reported for those of longer duration [17, 33]. 

 According to Apel et al. [11], exercise therapy is the most frequent CAM used in physiotherapy, which is an important part of rehabilitation. Vitamins, minerals, and other supplements are often added to exercise therapy. No effects or only slight improvements in MS symptoms have been reported for electrotherapy, gemstone therapy, hematogen oxidation therapy, homeopathy, psychotherapy, or oxygen therapy [6]. The most frequently reported benefits of CAM therapies are relaxation, improved sleep, pain reduction, spasm reduction, muscle strength, mobility, and general well-being [5, 42]. Relaxation techniques, massage, and Feldenkrais methods have all been associated with health benefits [11]. 

 We may have obtained better outcomes if individual Ai-Chi sessions had been offered, increasing the motivation by allowing the patient to select the music. Study limitations include the absence of an Ai-Chi group without ambient music or the presence of a control relaxation group with ambient music to explore the contribution of this element. Furthermore, although the experimental and control sessions were held on different days of the week, we cannot guarantee that participants were blinded to the nature of their group because they were all members of the same association (AEMA), which may have favored an overestimation of the effects of the Ai-Chi program.

## 5. Conclusions

According to these results, a 20-week Ai-Chi aquatic exercise program produces a significant pain reduction in MS patients that lasts for 10 weeks after the end of the program. It also improves other MS-related symptoms, including disability, depression, and fatigue. These effects of the Ai-Chi aquatic program were superior to those of an equivalent exercise program in a therapy room.

## Figures and Tables

**Table 1:** Demographic data at baseline.

	Experimental group	Control group	*P* value
Sex	26 females 10 males	24 females 13 males	0.312
Mean age: years (SD)	46 (9.97)	50 (12.31)	0.904
Age range: years	25–75	29–75	
Expanded Disability Status Scale: mean (SD)	6.3 (0.8)	5.9 (0.9)	0.723
Years since diagnosis: mean (SD)	10.7 (9.1)	11.9 (8.7)	0.915
Type of MS (*n*)			
Primary Progressive	6	9	0.425
Secondary Progressive	9	12	0.406
Not known	21	16	0.318
Pain VAS score: Mean (SD)	8.3 (1.2)	7.8 (1.6)	0.939

*P *value <0.05 (95% confidence interval).

**Table 2:** Median values and standard deviation values of outcome measures at each time point.

Outcome measure	Group allocation	Baseline	Week 20	Week 24	Week 30	Percentage change from baseline to week 20
Pain VAS	Control	7 (1.9)	6 (2.3)	6 (2.1)	6 (2.4)	23% Improvement
	Experimental	7 (2.1)	3 (2.3)^ ∗ *φ*^	4 (2.6)^ ∗ *φ*^	5 (2.5)*	50% Improvement

McGill Pain Questionnaire PRI	Control	23 (10.21)	20 (12.47)	21 (11.53)	22 (10.06)	17% Improvement
	Experimental	19 (11.34)	12 (7.45)^ ∗ *φ*^	14 (10.04)^ ∗ *φ*^	19 (12.19)	40% Improvement

McGill Pain Questionnaire PPI	Control	2 (1.5)	2 (1.1)	2 (1.4)	2 (1.3)	5% Improvement
	Experimental	2 (1.7)	1 (0.5)*	1 (1.5)	2 (1.8)	40% Improvement

Roland Morris Disability Questionnaire	Control	9 (6.11)	5 (4.27)*	6 (5.33)*	8 (5.91)	12% Improvement
	Experimental	7 (8.43)	2 (1.56)^ ∗ *φ*^	3 (2.32)^ ∗ *φ*^	3 (2.05)^ ∗ *φ*^	100% Improvement

Spasm VAS	Control	6 (3.1)	4 (4.5)	5 (3.86)	6 (2.76)	10% Improvement
	Experimental	5 (2.8)	2 (4.3)^ ∗ *φ*^	2 (3.9)^ ∗ *φ*^	4 (3.1)	91% Improvement

MSIS-29 Physical	Control	46 (18.34)	45 (17.14)	46 (19.12)	46 (15.93)	6% Improvement
	Experimental	48 (15.91)	41 (12.37)^ ∗ *φ*^	45 (11.25)^ ∗ *φ*^	48 (12.89)^ ∗ *φ*^	78% Improvement

MSIS-29 Psychological	Control	30 (23.53)	25 (19.36)*	27 (21.29)	29 (20.39)	37% Improvement
	Experimental	34 (29.47)	21 (15.73)^ ∗ *φ*^	22 (17.94)^ ∗ *φ*^	24 (11.27)^ ∗ *φ*^	81% Improvement

MFIS Physical	Control	25 (9.41)	22 (11.03)	23 (10.34)	24 (11.17)	9% Improvement
	Experimental	26 (9.02)	14 (10.37)^ ∗ *φ*^	17 (9.76)^ ∗ *φ*^	22 (13.81)	48% Improvement

MFIS Cognitive	Control	19 (8.95)	17 (7.13)	17 (8.59)	18 (10.27)	13% Improvement
	Experimental	23 (9.82)	13 (3.41)*	15 (6.28)*	17 (7.95)	61% Improvement

MFIS Psychosocial	Control	5 (2.8)	4 (3.1)	4 (2.9)	5 (3.4)	26% Improvement
	Experimental	5 (2.2)	2 (2.1)*	2 (1.3)*	3 (2.3)	58% Improvement

Fatigue Severity Scale	Control	5 (5.1)	4 (3.9)	5 (5.2)	5 (3.8)	12% Improvement
	Experimental	6 (3.1)	3 (2.2)*	3 (2.4)^∗ *φ*^	4 (2.2)	39% Improvement

Beck Depression Inventory II	Control	15 (8.68)	13 (5.91)	14 (9.01)	14 (8.93)	11% Improvement
	Experimental	14 (7.72)	5 (3.2)^∗ *φ*^	9 (4.88)^∗ *φ*^	11 (5.92)	52% Improvement

Barthel Index	Control	87 (10.34)	88 (8.92)	90 (7.65)	90 (8.73)	2% Improvement
	Experimental	91 (7.12)	86 (9.23)*	87 (8.79)*	89 (9.05)	9% Improvement

Median values and standard deviations (SD). *Significant change from baseline value. ^*φ*^Significant difference between experimental and control groups.
